# Using patient researchers to understand patient and family experiences in ICUs

**DOI:** 10.1186/cc14655

**Published:** 2015-03-16

**Authors:** H Stelfox, E McKenzie, S Bagshaw, M Gill, P Oxland, D Boulton, D Oswell, M Potestio, D Niven

**Affiliations:** 1 University of Calgary, AB, Canada; 2 University of Alberta, Edmonton, AB, Canada

## Introduction

With increasing emphasis on patient and family-centred care, it follows that patients and their family members should be included when priorities for improving care are established. We therefore used a novel methodology that employs former patients and family members as researchers to describe the experiences of critically ill patients and their families with ICUs and to identify opportunities for improvement.

## Methods

Using the patient engagement framework developed by Marlett and Emes, we engaged four former patients and family members trained in qualitative research methods to conduct and analyse semistructured focus groups and interviews with adult patients who had recovered from critical illness and family members of both surviving and deceased patients. Participants were recruited from 13 ICUs in Alberta, Canada. Focus groups and interviews were recorded, transcribed and analysed using phenomenological reduction. Data collection continued until thematic saturation was reached.

## Results

Thirty-two participants including patients (*n *= 11) and family members (*n *= 21) participated in five focus groups (*n *= 23 participants) and eight interviews (*n *= 9 participants). Participants articulated themes reflecting important components of care organised across three phases of the ICU experience; admission to ICU, daily care in ICU and after ICU discharge. Admission to ICU comprised three themes: patient and family transition into ICU, patient and family disorientation upon admission to ICU and preferred staff actions to help patients/ family adapt to the ICU. The daily care phase of ICU consisted of five themes: honouring patient's voices, needing to know, making decisions, culture in ICU and medical care. The experience after ICU discharge comprised two themes: transition from ICU to a hospital ward and long-term effects of critical illness. Participants identified five priorities for improvement: provide families with a guide/navigator; educate providers about the fragility of family trust; improve provider communication skills; inform patients about the long-term effects of critical illness; and develop strategies to facilitate continuity of care between providers.

## Conclusion

Patients and family members are an untapped resource and engaging them as researchers is a viable strategy to identify opportunities for quality improvement that are patient and family centred.

